# Refuges and host shift pathways of host-specialized aphids *Aphis gossypii*

**DOI:** 10.1038/s41598-017-02248-4

**Published:** 2017-05-17

**Authors:** Xiang-Dong Liu, Ting-Ting Xu, Hai-Xia Lei

**Affiliations:** Department of Entomology, Nanjing Agricultural University, Nanjing, 210095 China

## Abstract

Polyphagous cotton-melon aphid populations usually comprise cotton- and cucurbit-specialized biotypes. Host-specialized aphids are prone to food shortages. Cucumber, the favourite food of cucurbit-specialized aphids, is usually absent during autumn and winter in Nanjing, China. Therefore, suboptimal host plants act as refuges and govern the population dynamics of this aphid. The species, growth stages and leaf ages of host plants that cotton- and cucurbit-specialized aphids potentially could use were explored in this study. Cotton-specialized aphids were found to use wild chrysanthemum, potato, zucchini, pumpkin and flowering cucumber besides cotton, whilst cucurbit-specialized aphids were able to utilize potato, zucchini, pumpkin and mature cotton besides cucumber. The population dynamics and genotype frequencies of aphids on hibiscus, cotton, zucchini, cucumber and pumpkin showed that cotton-melon aphids on cucumber could transfer onto mature cotton. Aphids on zucchini shared microsatellite genotypes with aphids on cotton and cucumber. The predominant genotype of aphids on cotton was found on hibiscus, but the predominant genotype on cucumber was not found on hibiscus. Host-specialized aphids clearly have refuges during food shortages. Hibiscus is an overwintering host for cotton-specialized aphids but not for cucurbit-aphids. Removing refuges or managing aphids on refuges could potentially be an effective method to control cotton-melon aphids.

## Introduction

Host plants play a key role in the population dynamics of phytophagous insects^1–4^. Insect survival, development and fecundity are strongly dependent on their host plants^5, 6^. For example, the survival of the red cotton bug (*Dysdercus cingulatus* (Fab.)) (Hemiptera: Pyrrhocoridae) was very low when feeding on Indian mallow (*Abutilon indicum* L.), low on coastal hibiscus (*Hibiscus tiliaceus* L.), and intermediate on musk mallow (*Abelmoschus moschatus* Medik) (all Malvales: Malvaceae)^3^. Similarly, *D. cingulatus* developed faster when fed cultivated crops than when fed wild plants^3^, whilst the reproductive rate of the bird cherry-oat aphid (*Rhopalosiphum padi* (L.)) (Hemiptera: Aphididae) was the highest on oat (*Avena sativa* L.) and wheat (*Triticum aestivum*) and low on the grasses *Phleum pratense* L. and *Dactylis glomerata* L. (Poales: Poaceae)^5^. The pea aphid (*Acyrthosiphon pisum* Harris) was found to perform better on broad bean (*Vicia faba* L.) and alsike clover (*Trifolium hybridum* L.) (Fabales: Fabaceae), with the greatest population size, least time to maturity and lowest mortality, but did not survive to reproduce on white clover (*Trifolium repens* L.), common restharrow (*Ononis repens* L.), Scotch broom (*Sarothamnus scoparius* (L.)) or vetch (*Vicia cracca* L.) (Fabales: Fabaceae)^6^. The fecundity of the spirea aphid (*Aphis spiraecola* Patch) was higher on the chicken-gizzard aralia (*Polyscias crispata* (Bull)) (Apiales: Araliaceae) than on rough lemon (*Citrus jambhiri* Lushington) and pineapple orange (*C. sinensis* (L.)) (Sapindales: Rutaceae), and the developmental time for the immature stages was shorter^7^. Clearly, host plants affect the population dynamics of insects.

In the case of aphids, the growth stage and structure of host plants also affect performance. For example, the intrinsic rate of increase of the green peach aphid (*Myzus persicae* (Sulzer)) was higher on chrysanthemum during the flowering stage than during the vegetative period^8^. Generally, cotton-melon aphids (*Aphis gossypii* Glover) are observed with greater frequency on the upper and middle parts of cotton plants^9^. Higher numbers of *A. gossypii* were found on positions 1 and 2 of vegetative and fruiting branches than on the other branch positions in cotton^10^. The cotton-melon aphid also preferred the upper parts of chrysanthemum over the bottom parts of the plant^8, 11^. However, the insects preferred the bottom, older leaves of eggplant^12^. Both the aphid *M. persicae* and the potato aphid (*Macrosiphum euphorbiae* Thomas) performed better on developmentally young potato plants than on mature ones^13^. The nutritional quality of a host plant for aphids is dependent on the growth stage of the plant. The amino acid composition of the phloem in potato has been found to vary significantly with time, which shapes the nutritional quality of plants and consequently determines the performance of aphids^13^.

The cotton-melon aphid is widely distributed in tropical, subtropical and temperate regions. In China, the life cycle of this aphid is generally holocyclic and heteroecious. The aphids mainly overwinter on woody plants (primary host), such as hibiscus (*Hibiscus syriacus* L.), with diapausing eggs and immigrate into cotton fields during late spring and early summer after undergoing three to four generations on the primary host through parthenogenesis. In late autumn, they produce sexual forms and return to the primary host plants to mate and lay eggs^14^. Additionally, an anholocyclic or monoecious life cycle has also been found in cotton-melon aphid populations on some specific host plants, such as hibiscus and cucumber^14–17^. Cotton-melon aphids mainly damage cotton and cucurbit crops by direct sap feeding and by acting as vectors of various pathogenic plant viruses. They can feed on 912 species plants belonging to 116 families^18^. However, a population occurring on a particular species of host plant can usually utilize only a subset of the host range^2, 19–21^. For example, *A. gossypii* on cotton cannot use cucumber, and aphids on cucumber cannot use cotton^2, 22^. Cucurbit- and cotton-specialized *A. gossypii* biotypes or races have been found in many countries^21, 23–26^. In China, *A. gossypii* populations on cotton and cucumber cannot reciprocally transfer, but they can use zucchini and cowpea^20, 22, 25^.

The growing period of cucurbit crops, such as cucumber and zucchini, is much shorter than that of cotton. Generally, cucumber crops senesce in summer. During the autumn and winter, there are often no cucumber crops in natural fields. Consequently, a big problem for cucurbit-specialized aphids on cucumber is where they can go when cucumber host plants are unavailable. Therefore, in the present study, we aimed to explore the potential host plants for host-specialized aphids and to determine which plants and crops would act as transitional refuges or bridges during host shifts in host-specialized aphids. We also examined the roles of growth stage and leaf nutritional quality of host plants in acting as refuges for the cotton- and cucurbit-specialized aphid *A. gossypii*. The results would be helpful in exploring the possible host transfer pathways of cotton-melon aphids and provide an effective method for managing this aphid in cotton and cucurbit fields based on these refuge plants.

## Materials and Methods

### Aphids

Two host-specialized biotypes of the cotton-melon aphid (*A. gossypii*), cotton- and cucurbit-specialized aphids, were reared asexually in the laboratory for approximately 10 years on cotton and cucumber seedlings, respectively^22, 27^. The cotton-specialized (CO) and cucurbit-specialized aphids (CU) were originally collected from cotton and cucumber fields, respectively, in Nanjing, China.

### Fitness of CU and CO on different host plants

The population dynamics of CO and CU on seven plants, wild chrysanthemum (*Chrysanthemum indicum* L.) (Asterales: Asteraceae), potato (*Solanum tuberosum* L.) (Solanales: Solanaceae), tomato (*Solanum lycopersicum* L.), radish (*Raphanus raphanistrum* (L.)) (Brassicales: Brassicaceae), maize (*Zea mays* L.) (Poales: Poaceae), pumpkin (*Cucurbita moschata* Duchesne), and zucchini (*Cucurbita pepo* L.) (Cucurbitales: Cucurbitaceae), were investigated under laboratory conditions at 27 °C and 75% RH. Wild chrysanthemums were collected from the campus of Nanjing Agricultural University, Weigang, Nanjing, and 10–15 cm high seedlings were used for rearing aphids. The other six crops were cultured using seeds in plant growth chambers at 27 °C. The stems or leaves cut from plants were used to rear CO and CU. Stems and leaf petioles were wrapped in wet cotton wool to keep them fresh. A stem or leaf was placed into a Petri dish (90 mm diam., 20 mm height), and then 15 5-day-old apterous aphids were released into the dish. The total number of aphids in a Petri dish was investigated every two days for 20 days after aphid released. The experiment using CU and CO on each species of host plant was performed with 30 replicates. If all aphids in a replicate died, the maximum longevity of this population was recorded. Due to the peak in the population size of CU and CO occurring on different days after aphid released, the maximum growth rate over 20 days was computed by the ratio of the population size at its peak to the number of aphids originally released (15 aphids). We used the maximum longevity and growth rate of the populations to assess the fitness of CU and CO on different host plants.

### Population growth of CO on the seedling and flowering stages of cucumber

To determine whether the growth stage of a host plant could affect the host use of host-specialized aphids, the population growth of CO on the seedling and flowering stages of cucumber was studied. Cucumber seedlings were planted in pots (85 mm diam., 120 mm height) under laboratory conditions (27 °C and 75% RH). When the cucumber plants were flowering, all flowers on five plants were removed, and the other five plants remained intact. The cucumber plants at the flowing stage with and without flowers and at the seedling stage with four leaves were used for rearing CO. Ten five-day-old CO were translocated onto each of these three types of cucumber plants, and the plants were then covered by net cages. The total number of aphids on a plant was observed and recorded daily. If CO could survive for 40 days on a specific stage of cucumber and their population size increased, we concluded that this growth stage was suitable for CO. The experiment for each cucumber plant type was initially replicated five times, but two replicates using cucumbers with cut flowers unfortunately failed because of an accident that occurred during the observations.

### Population growth of CU on different stages of cotton plants

We also examined the ability of CU to use young and older cotton. Cotton plants grown in plastic pots (diam. 85 mm and height 120 mm) at 27 °C in a growth chamber were divided into two groups when they had four true leaves. One group was watered with 100 ml of a liquid nutrient solution per pot per week (normal nutrients) in which the plants grew vigorously with tender and green leaves, named ‘vigorous cotton’, whilst the other group was given half the amount of these nutrients, 50 ml per week (poor nutrient), in which the plants grew poorly with small-sized yellowish leaves, named ‘poor cotton’. When these two groups of cotton had six mature leaves, they were used for rearing CU. On the other hand, cotton plants grown for six months with 18–22 mature leaves under normal nutrient conditions in a chamber named ‘old cotton’ were also used in this experiment. During the experiment, ten 5-day-old CU were translocated onto a cotton plant, which was covered with a net cage. Then, the population size of aphids on a plant was checked daily for 20 days. The experiments for CU on vigorous and poor cotton were replicated nine times, and the experiment involving CU on old cotton was replicated three times.

### Fitness of CU on different leaves of a cotton plant

This experiment aimed to determine the ability of CU to use different leaves on a cotton plant. Cotton plants were cultivated in pots under laboratory conditions as above. Due to the limitations of nutrients and space in a pot, the cotton grown over a period of six months had only 18–22 leaves and no fruiting branches. The cotton leaves on the basal part of a plant were older and yellowish, whilst leaves on the middle and upper parts were young and green. Therefore, we designated the first six leaves from the basal part of the cotton plant as the ‘basal leaves’, the second six leaves following the basal leaves as the ‘middle leaves’, and the other six leaves following the middle leaves as the ‘upper leaves’. These three leaf types were cut from the cotton plants, the petioles were wrapped in wet cotton wool to keep the leaves fresh, and then the leaves were placed in Petri dishes (90 mm diam., 20 mm height) to rear CU. Ten five-day-old apterous CU were translocated onto a basal, middle or upper leaf in a Petri dish. The number of aphids per dish was checked daily for 19 days. The experiments involving CU reared on the basal, middle and upper cotton leaves each included 12 replicates.

At the same time, we also used cotton plants with 10 leaves (78 days old) to examine the ability of CU to use different cotton leaves using the life table method. Nine cotton leaves with the exclusion of new leaves that were not fully expanded were removed and categorized into three leaf types: basal leaves (3 leaves), middle leaves (3 leaves), and upper leaves (3 leaves), based on the position of the leaves on the plant. In total, 81 leaves were collected from nine plants. The petioles of the excised leaves were wrapped in wet cotton wool to keep them fresh. An excised cotton leaf placed in a Petri dish was used to rear CU. Ten apterous adult CU were translocated onto a leaf to produce offspring to establish a life table. If five or more offspring were produced in one day, the adult aphids were removed, and these newborn aphids were considered to be the original cohort for the life table. Leaves on which the 10 adult aphids could not produce five offspring in a day were removed. Life tables for CU on 23 upper, 12 middle and 12 basal leaves were established. The observations necessary to produce a life table were performed every two days. When the original cohort started to reproduce, their offspring were counted and removed. The observations were discontinued when the original cohort died.

### Population dynamics of cotton-melon aphids on different host plants in the field

Cotton, zucchini, cucumber and pumpkin were cultured in 2 m × 2 m plots (6 plants per plot) in Nanjing, China. Only one crop species was planted in each plot, and all four species of crops were distributed evenly among 16 plots. There was a 30 cm wide ditch between two adjacent plots. The crops were planted on 24 May, 2013. The population survey of cotton-melon aphids on each plant was carried out once a week during June to December. All the cucumber plants died on 22 September, and zucchini and pumpkin died on 17 November. Only the cotton plants survived after the last survey. The number of cotton-melon aphids on all the plants was observed by the naked eye. From 12 August to 27 October, 15-32 aphid samples were collected from each crop to examine their genotypes (see below), and only 1-2 aphids were collected from each plant during each sampling event. This sampling did not ultimately affect the population dynamics of the aphids in the field.

### Microsatellite genotyping of cotton-melon aphids on different host plants in the field

The DNA from one aphid was extracted according to the method of Zhang *et al*.^28^. Eight microsatellite loci, Ago-24, Ago-53, Ago-59, Ago-66, Ago-69, Ago-84, Ago-89 and Ago-126, were chosen to genotype each aphid using the PCR method^29^. The PCR reactions were conducted in a 12.5 μL volume containing 1 μL DNA template, 6.25 μL 2 × MightyAmp buffer (Mg^2+^, dNTP plus), 0.5 μL forward and reverse primers (10 μM, Table S1), 0.5 μL MightyAmp DNA polymerase, and 3.75 μL double-distilled H_2_O. The PCR cycling parameters were 98 °C denaturation for 2 min followed by 35 cycles of 98 °C for 30 s and an annealing temperature of approximately 67 °C (based on Vanlerberghe-Masutti *et al*.^29^, Table S1) for 30 s, with a final extension step at 72 °C for 10 min. The amplification products were resolved in an 8% nondenaturating polyacrylamide gel and visualized using silver staining. The microsatellite band size was used to define aphid genotypes. Four genotypes were found among 738 aphid samples and named genotype I, II, III and IV in this study.

### Data analysis

A nonparametric Mann-Whitney U test was used to analyse the maximum longevity and growth rate of CO and CU on different host plants, as the data were not normally distributed. The aphid population sizes on different kinds of host plants were analysed using a mixed model for repeated-measures in GLM because the population sizes surveyed on different dates represented a repeated-measurement variable. The net reproductive rate (*R*_0_) and average generation time (*T*) were calculated using the life table method: *R*_0_ = ∑*l*_*x*_ *m*_*x*_ and *T* = ∑*xl*_*x*_ *m*_*x*_/∑*l*_*x*_ *m*_*x*_, respectively, where *l*_*x*_ is the proportion of individuals in the original cohort alive at age *x*, and *m*_*x*_ is the mean number of female progeny produced per female alive in age interval *x* ^27^. If aphids did not produce any progeny on a host plant, *T* was calculated by the weighted average method according to the longevity of each individual. The *R*_0_ and *T* of CU on the upper, middle and basal leaves of cotton were analysed using ANOVA followed the Tukey HSD post hoc comparisons method. These analyses were performed with SAS 9 software^30^.

## Results

### Host plants of cotton- and cucurbit-specialized aphids

The cotton-melon aphids had different capacities to use different host plants (Table 1). The cotton-specialized aphids in 16 out of 30 replicates could establish populations on chrysanthemum, but no cucurbit-specialized aphids used chrysanthemum. The maximum longevity of cotton-specialized aphids that could not establish a population on chrysanthemum was also significantly longer than that of the cucurbit-specialized aphids on chrysanthemum (U = 138.5, P = 0.018), and their maximum growth rates were also significantly higher (U = 129.5, P < 0.001). The cotton-specialized aphids used potato well, whereas of the cucurbit-specialized aphids, only 4 out of 30 replicates established populations on potato, and their growth rates were significantly lower than those of the cotton-specialized aphids (U = 122.0, P < 0.001). Both the cotton- and cucurbit-specialized aphids could hardly use tomato, radish and maize. All the cucurbit-specialized aphids could use pumpkin, and only a half of the cotton-specialized aphids could establish populations on pumpkin. The maximum growth rates of the cucurbit-specialized aphids on pumpkin were significantly higher than those of the cotton-specialized aphids (U = 60.0, P < 0.001). Both the cotton- and cucurbit-specialized aphids could use zucchini, and the maximum growth rates of cucurbit-specialized aphids were higher than those of cotton-specialized aphids (U = 234.0, P = 0.001). Chrysanthemum and potato were more suitable for cotton-specialized aphids than for cucurbit-specialized aphids, whereas pumpkin and zucchini were more suitable for cucurbit-specialized aphids (Table 1).

**Table 1:** Performance of cotton- and cucurbit-specialized aphids on different host plants.

Host plant	No. repli.	No. repli. failed		Maximum longevity of failed population (d)		Maximum growth rate of successful population during 20 days^a^	
		CO	CU	CO	CU	CO	CU
Chrysanthemum	30	16	30	14.0 ± 1.7*	8.9 ± 0.5	3.02 ± 0.33	0
Potato	30	0	4	NA	16.5 ± 0.5	9.41 ± 0.84**	4.62 ± 0.41
Tomato	30	30	30	2.9 ± 0.2 ns	2.7 ± 0.2	0	0
Radish	30	30	30	4.5 ± 0.3 ns	4.0 ± 0.3	0	0
Maize	30	30	30	2.0 ± 0.0 ns	2.0 ± 0.0	0	0
Pumpkin	30	16	0	14.4 ± 1.2	NA	2.77 ± 0.39**	4.92 ± 0.42
Zucchini	30	0	0	NA	NA	12.45 ± 0.87**	17.14 ± 1.02

^a^Successful population is defined here as that in which size was more than 2-fold of its original number after 20 days on a host plant.

### Effect of the growth stage of cucumber on cotton-specialized aphids

**Figure 1:**
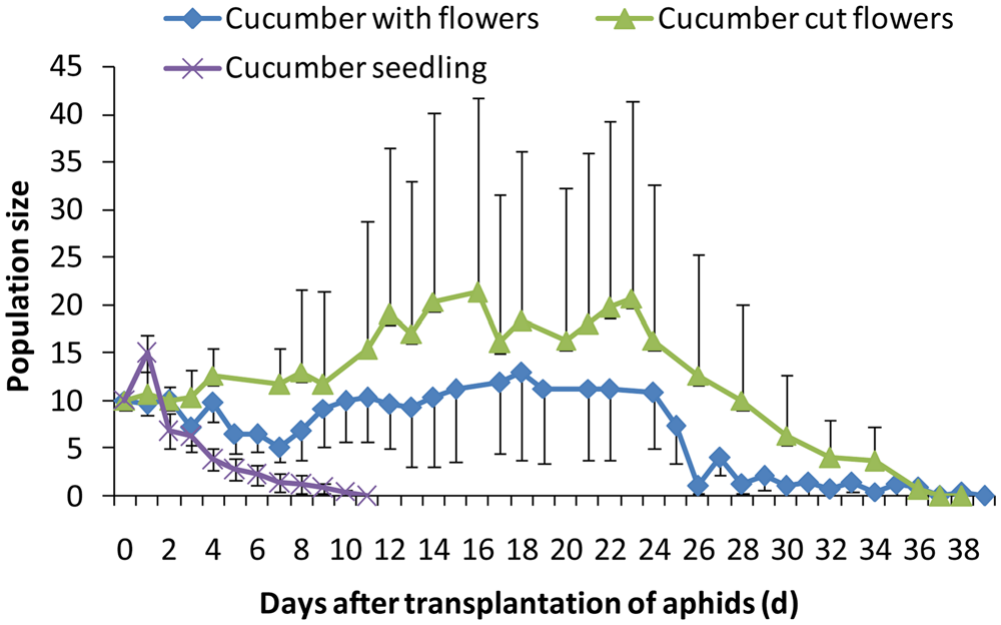
Population sizes (mean ± SE) of the cotton-specialized aphids on cucumber plants in the seedling and flowering stages.

The cotton-specialized aphids could not establish populations on cucumber in the seedling and flowering stages (Fig. 1). Although the cotton-specialized aphids survived 26 days longer on the flowering cucumber than on the those in the seedling stage, the population sizes of aphids on seedling and flowering cucumber were not significantly different (F_2, 10_ = 1.81, P = 0.2133, Fig. 1).

### Effect of the growth stage of cotton on cucurbit-specialized aphids

**Figure 2:**
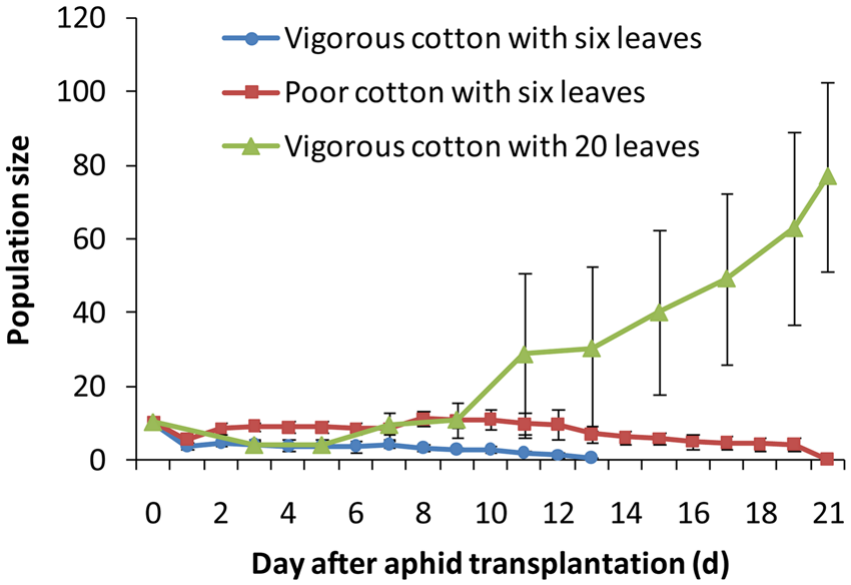
Population sizes (mean ± SE) of the cucurbit-specialized aphids on vigorous and poor seedling and old mature (6-month-old) cotton plants.

The cucurbit-specialized aphids could not establish populations on young seedlings of cotton regardless of whether vigorous or poor plants were used, although the population sizes of aphids were higher on the poor seedlings than on the vigorous seedlings for 20 days after aphid translocation (F_1, 16_ = 21.304, P < 0.001). However, the cucurbit-specialized aphids could establish populations on mature cotton plants with 20 leaves, and the population size was significantly higher than that on vigorous and poor seedlings (F_2, 18_ = 6.69, P = 0.0067, Fig. 2).

### Effect of the leaf age of cotton on cucurbit-specialized aphids

**Figure 3:**
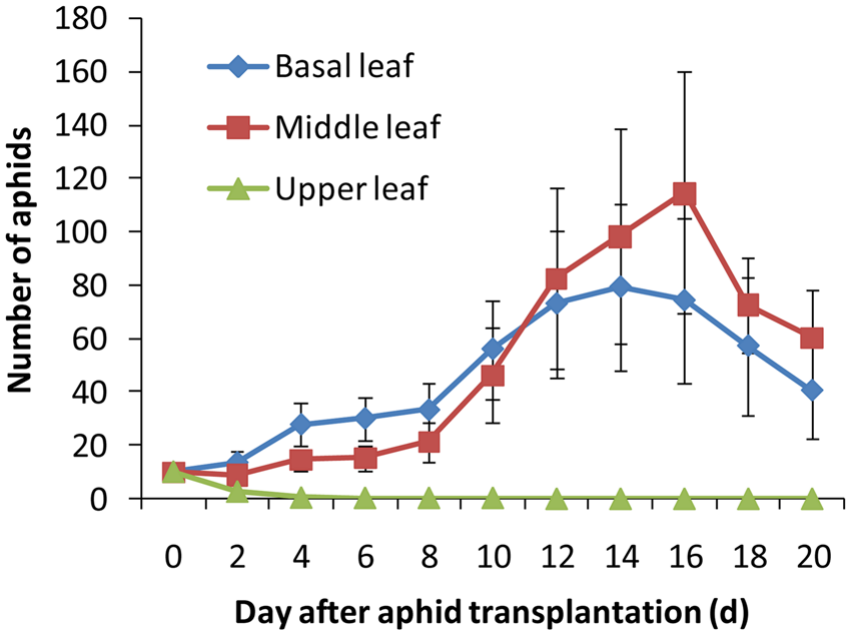
The number of cucurbit-specialized aphids (mean ± SE) on upper, middle and basal leaves from 6-month-old cotton plants.

**Figure 4:**
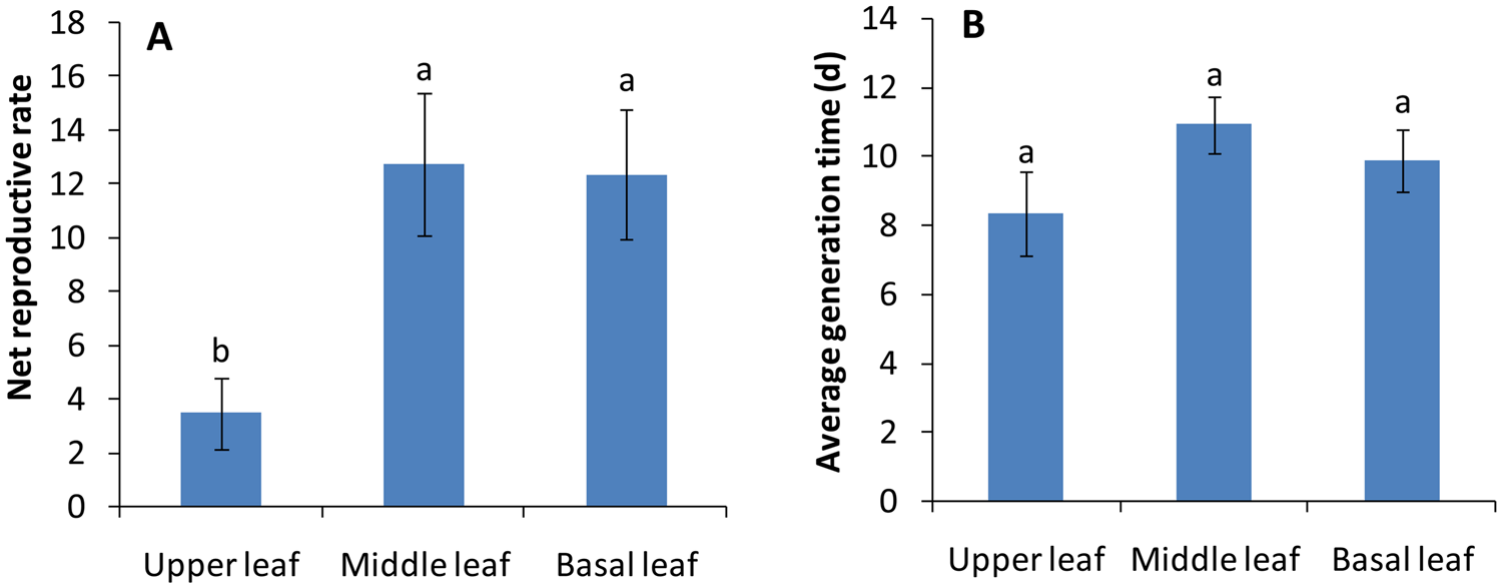
Net reproductive rate (**A**) and average generation time (**B**) of cucurbit-specialized aphids on upper, middle and basal leaves of cotton seedlings with 10 leaves (mean ± SE).

The population sizes of cucurbit-specialized aphids reared on the upper young leaves of cotton declined quickly, and all aphids died after four days. However, on the middle and basal older leaves, the population sizes increased in the first 14–16 days, and then the population sizes decreased as the leaves became withered (Fig. 3). Overall, the population sizes of cucurbit-specialized aphids on the middle and basal leaves of cotton were significantly higher than that on the upper leaves (F_2_, _24_ = 5.33, P = 0.0121, Fig. 3). Similarly, the net reproductive rates of cucurbit-specialized aphids on the middle and basal old leaves of cotton were significantly higher than that on the upper young leaves (F_2, 44_ = 8.292, P = 0.001), although the average generation time did not differ among different leaves (F_2, 44_ = 1.283, P = 0.287, Fig. 4).

### Population dynamics and genotypes of the cotton-melon aphids on different host plants in the field

**Figure 5:**
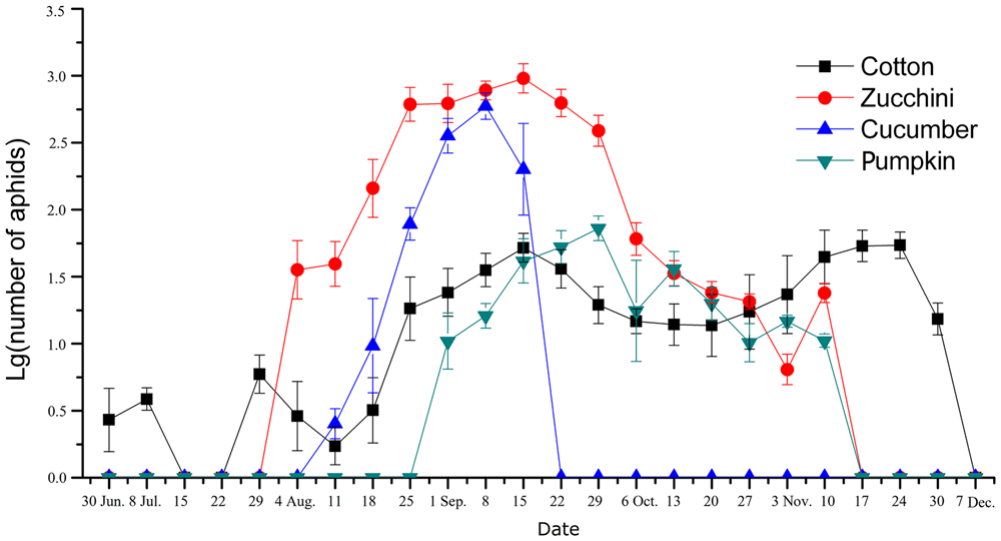
Population dynamics of cotton-melon aphids on intercropped cotton, zucchini, cucumber and pumpkin in 2013 (mean ± SE).

In an intercropping field with cotton, zucchini, cucumber and pumpkin crops, the cotton-melon aphids were initially found on cotton, then on zucchini and cucumber, and later on pumpkin. When the population size of aphids on cucumber decreased on 15 September due to withered leaves, the number of aphids on pumpkin, cotton and zucchini increased. As zucchini and pumpkin became withered after 13 October, the population sizes of aphids on these species decreased, but that of aphids on cotton increased (Fig. 5). This result implies that aphids on cucumber might transfer onto other cucurbits and cotton when encountering a food shortage.

**Figure 6:**
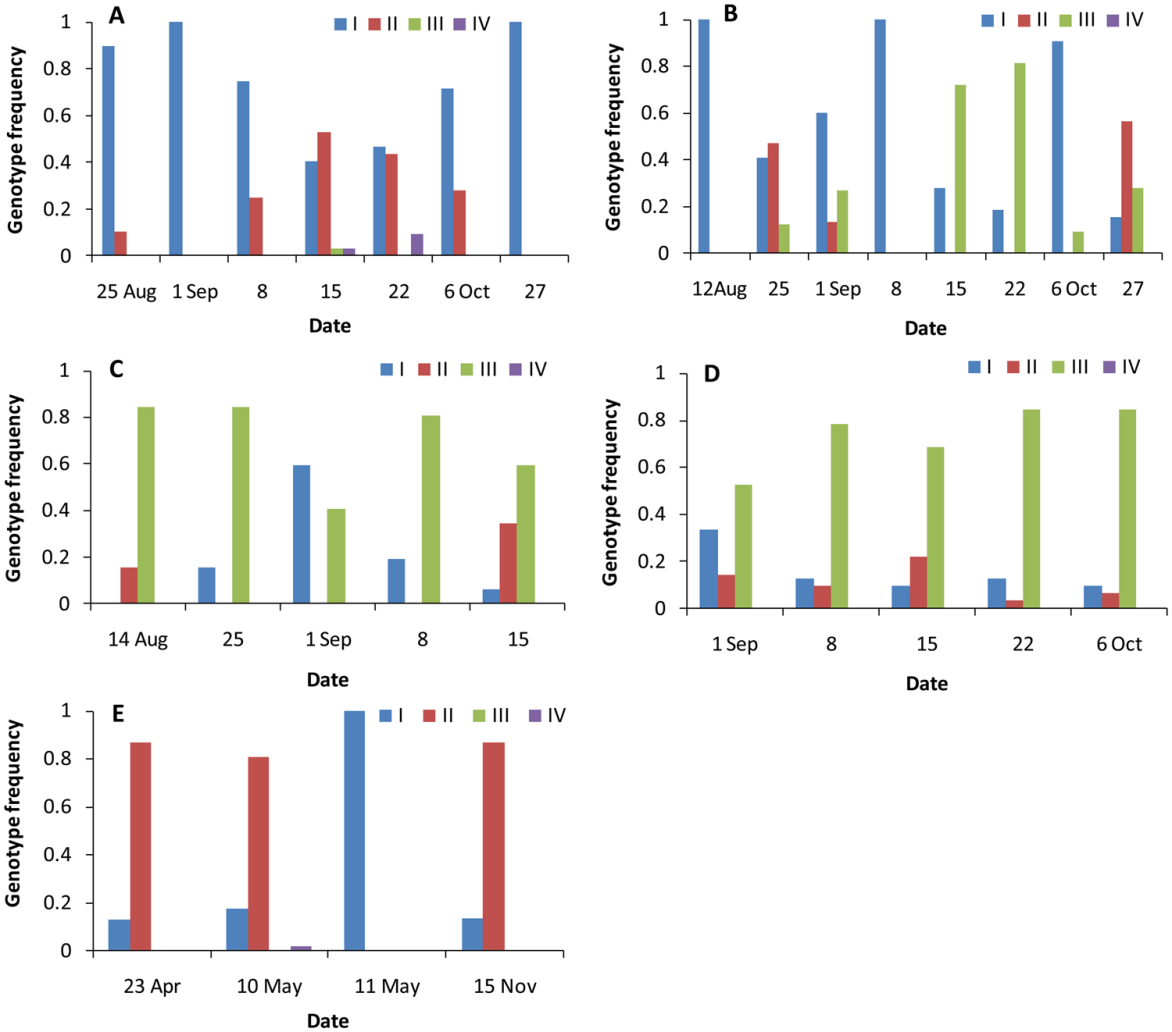
Genotype frequencies of *Aphis gossypii* on cotton (**A**), zucchini (**B**), cucumber (**C**), pumpkin (**D**) and hibiscus (**E**) on different survey dates in 2013.

The predominant genotype of aphids on cotton was genotype I (74.86%, Fig. 6A), and genotype III was predominant among aphids on cucumber (69.90%, Fig. 6C) and pumpkin (73.60%, Fig. 6D). Although genotypes I-IV were found in the aphid populations on cotton, the genotypic frequencies of III and IV were very low and only found on 15 and 22 September, when the cucumber crops withered. The genotypes III and IV on cotton would transfer from cucumber (Fig. 6A). The genotypes of aphids on zucchini belonged to genotypes I, II and III, and the predominant genotype in the populations varied on different survey dates (Fig. 6B). This suggests that zucchini can act as a refuge for cotton- and cucurbit-specialized aphids. On an overwintering plant, hibiscus, genotypes I, II and IV were found but not genotype III (Fig. 6E). The aphids on cucumber might not transfer onto hibiscus to overwinter.

## Discussion

### Refuges of host-specialized aphids

The suboptimum host plants provide refuges for host-specialized aphids. We found that only part of the cotton-specialized aphids could survive and reproduce on wild chrysanthemum and cultivated pumpkin. This suggests that the wild chrysanthemum growing during all seasons and pumpkin cultivated in parallel with cotton can provide refuges for cotton-specialized aphids during periods of a food shortage. The cotton-melon aphids on chrysanthemum were able to produce sexual females and males under naturally occurring short-day conditions^19^. This finding implies that aphids taking refuge on chrysanthemum would successfully complete a holocyclic life cycle in a year and overwinter as eggs from sexual reproduction. On the other hand, all the cotton-specialized aphids could establish populations on potato and zucchini, although the survival and fecundity were lower in the first generation^20, 22^. Potato and zucchini would act as suitable host plants for cotton-specialized aphids. However, potato grows only in winter and spring in Nanjing. Therefore, it would be a refuge for cotton-specialized aphids before they migrate to cotton. The growing period of zucchini is similar to that of cotton, and cotton-specialized aphids perform well on it^20, 22^. This indicates that zucchini is a suitable host plant or refuge for cotton-specialized aphids. The genotypes of cotton-melon aphids on zucchini and cotton examined in this study and by Wang *et al*.^31^ also support this result because the genotypes of aphids on zucchini were similar to those on cotton. In this study, we also found that cotton-specialized aphids could survive for only 10 days on cucumber seedlings but could survive for more than one month on the flowering stage of cucumber. We suggest that flowering cucumber could prove to be a transitional refuge for cotton-specialized aphids during food shortages.

Cucurbit crops, such as cucumber, zucchini, watermelon, muskmelon and hami melon, are the main hosts of cucurbit-specialized aphids^14, 32^. In this study, we found that some cucurbit-specialized aphids could use potato but hardly ever use chrysanthemum. *Aphis gossypii* on potato could transfer onto cucumber and zucchini^33^. Cucurbit-specialized aphids rarely produced sexual forms under natural short photoperiods and low temperatures^14, 34^ and might overwinter as virginoparae in warm habitats, such as in glasshouses. Potato grows earlier than cucurbit crops in Nanjing and might be an alternative host plant for the cucurbit-specialized aphids when the cucurbit crops are not available.

Interestingly, we found that cucurbit-specialized aphids could use the old leaves of cotton and established populations on the over 6-month-old mature cotton plants but that they could not use seedlings of cotton or new, tender leaves. We presume that the cucurbit-specialized aphids choose mature cotton as a refuge in summer and autumn when the cucurbits are withered. The fact that genotype III, i.e., cucurbit-specialized aphids, were present on cotton when the cucumber crops died largely supported our assumption. Our previous study showed that cucurbit-specialized aphids reared on whitefly-stressed old cotton for 61 days still maintained a strong preference for their natal host, cucumber^27^. Therefore, the mature cotton plants only acted as a refuge for cucurbit-specialized aphids when cucurbit crops were not available.

### Host shift pathways of cotton-melon aphids

Based on the potential refuges of cotton- and cucurbit-specialized aphids, the cotton-melon aphids on cotton mainly derive from overwintering primary hosts, such as hibiscus (*H. syriacus*) (Malvales: Malvaceae), pomegranate (*Punica granatum* L.) (Myrtales: Lythraceae) and Chinese prickly ash (*Zanthoxylum simulans* Hance) (Sapindales: Rutaceae)^31, 33, 35^, and a part of them might be from wild chrysanthemum or the other host plants. The cotton-melon aphids on primary hosts can transfer onto zucchini^22, 32^. During summer, aphids could transfer between cotton and zucchini^14^. Our previous study showed that when cotton-specialized aphids were reared on zucchini for more than five generations, they would lose the capacity to use cotton^22^. Therefore, the transfer of cotton-specialized aphids between cotton and zucchini was not always bidirectional.

The cucurbit-specialized aphids on cucumber do not directly come from an overwintering primary plant of cotton-specialized aphids such as hibiscus because the aphids on hibiscus could not establish populations on cucumber^2^. The finding that aphids occurred later on cucumber than on cotton in the field implies that the sources of *A. gossypii* settling on cucumber are different from those settling on cotton. *Aphis gossypii* on potato and other cucurbit crops, such as zucchini, might be the sources of aphids on cucumber. Aphid populations could exchange freely among the cucurbit crops cucumber, zucchini and pumpkin in summer. When the cucurbit crops become withered, the cucurbit-specialized aphids would take refuge on mature cotton and then transfer onto an overwintering host. The cucurbit crops in glasshouses and greenhouses were considered to be overwintering hosts for cucurbit-specialized aphids^14^.

### Maintenance of host specialization in cotton-melon aphids

Host specialization is an obvious characteristic of *A. gossypii* populations in the field, although the cotton- and cucurbit-specialized aphids can share the summer hosts zucchini and cowpea^20, 22, 31^. The differences in the predominant aphid genotypes on cotton and cucumber showed that host specialization in *A. gossypii* indeed exists in the field. The coexistence of three genotypes on zucchini suggests that both cotton- and cucurbit-specialized aphids could transfer onto zucchini. Will cotton-melon aphids change their host specialization after they share the same host plant? We think that the answer might be no because cucurbit-specialized aphids could not produce sexual morphs regardless of the host plant they fed on, and their life cycles were anholocyclic, but cotton-specialized aphids would produce sexual morphs before overwintering, and their life cycles were holocyclic^14, 34^. In southern France, *A. gossypii* populations on the cucurbits zucchini, melon and cucumber were primarily asexual^15^. Therefore, gene flow between cotton- and cucurbit-specialized aphids was prevented. The host plants zucchini and potato only provided refuges for cotton- and cucurbit-specialized aphids to avoid food shortages or poor habitats. After living on refuges, the host-specialized aphids would return to their native host plants to complete their relatively consistent life cycle. Hence, host specialization could be maintained.

The maintenance of host specialization restricts gene flow among cotton-melon aphids on different host plants. The genotype variations of the tansy aphid (*Metopeurum fuscoviride* Stroyan) have been found to be determined by the essential oil composition of the tansy plants themselves^36^. The different genotypes of *A. gossypii* found on different host plants might have resulted from limited gene flow. A polyphagous population was usually found to comprise cryptic species, subspecies or biotypes that could use different host plants^37, 38^. It is possible that the cotton-melon aphid populations are indeed composed of cryptic species based on this and previous studies^34^, although no differences in the *COI* gene sequence were found between the cotton- and cucurbit-specialized aphids^39^. In light of this, more high-resolution molecular markers, especially direct DNA sequencing of the genome, are required to further elucidate the host-dependent genetic variations of this aphid.

Cotton-melon aphids have evolved high-level resistance to multiple insecticides^40, 41^, which has resulted in control failures or low control effectiveness. Based on the refuges and host shift pathways of cotton-melon aphids, removing refuges or managing the aphids in refuges might be an effective method for controlling this aphid. For example, in regions where cotton and cucumber crops are grown, if zucchini and potato are planted less or not at all, the cotton-melon aphid populations will naturally decrease on cucumber. If aphids are controlled well on limited and concentrated overwintering host plants before migration to cotton, population outbreaks of the cotton-melon aphids on cotton will become relatively rare, or so we assume.

### Ethical approval

This article does not contain any studies with human participants performed by any of the authors.

## Electronic supplementary material

Supplemental Material
