# Supplementary material for: Refuges and host shift pathways of host-specialized aphids Aphis gossypii
Source: Sci Rep. 2017 May 17;7:2008. doi: 10.1038/s41598-017-02248-4 (PMC5435715; doi:10.1038/s41598-017-02248-4)
Supplement: Supplementary file 1 — Supplemental Material [file 41598_2017_2248_MOESM1_ESM.pdf]

Refuges and host shift pathways of host-specialized aphids *Aphis gossypii*

Xiang-Dong Liu*, Ting-Ting Xu, Hai-Xia Lei

Department of Entomology, Nanjing Agricultural University, Nanjing 210095, China

* Correspondence: liuxd@njau.edu.cn. Tel:+86-25-84395242

Locus	Primer sequence (5'-3')	Annealing temperature (^{o}C)
Ago-24	TTTTCCCGGCACACCGAGT	67
	GCCAAACTTTACACCCCGC	
Ago-53	TGACGAACGTGGTTAGTCGT	67
	GGCATAACGTCCTAGTCACA	
Ago-59	GCGAGTGGTATTCGCTTAGT	67
	GTTACCCTCGACGATTGCGT	
Ago-66	TCGGTTTGGCAACGTCGGGC	67
	GACTAGGGAGATGCCGGCGA	
Ago-69	CGACTCAGCCCCGAGATTT	65
	ATACAAGCAAACATAGACGGAA	
Ago-84	GACAGTGGTGAGGTTTCAA	60
	ACTGGCGTTACCTTGTCTA	
Ago-89	GAACAGTGCTCGCAGTCTAT	67
	GACAGCGTAAACATCGCGGT	
Ago-126	GGTACATTCGTGTCGATTT	62
	TAAACGAAAAAACCACGTAC	

Table S1. Primer sequence of microsatellite.
